# Supplementary figures and images for: Myosin V executes steps of variable length via structurally constrained diffusion
Source: eLife. 2020 Jan 15;9:e51569. doi: 10.7554/eLife.51569 (PMC7054003; doi:10.7554/eLife.51569)

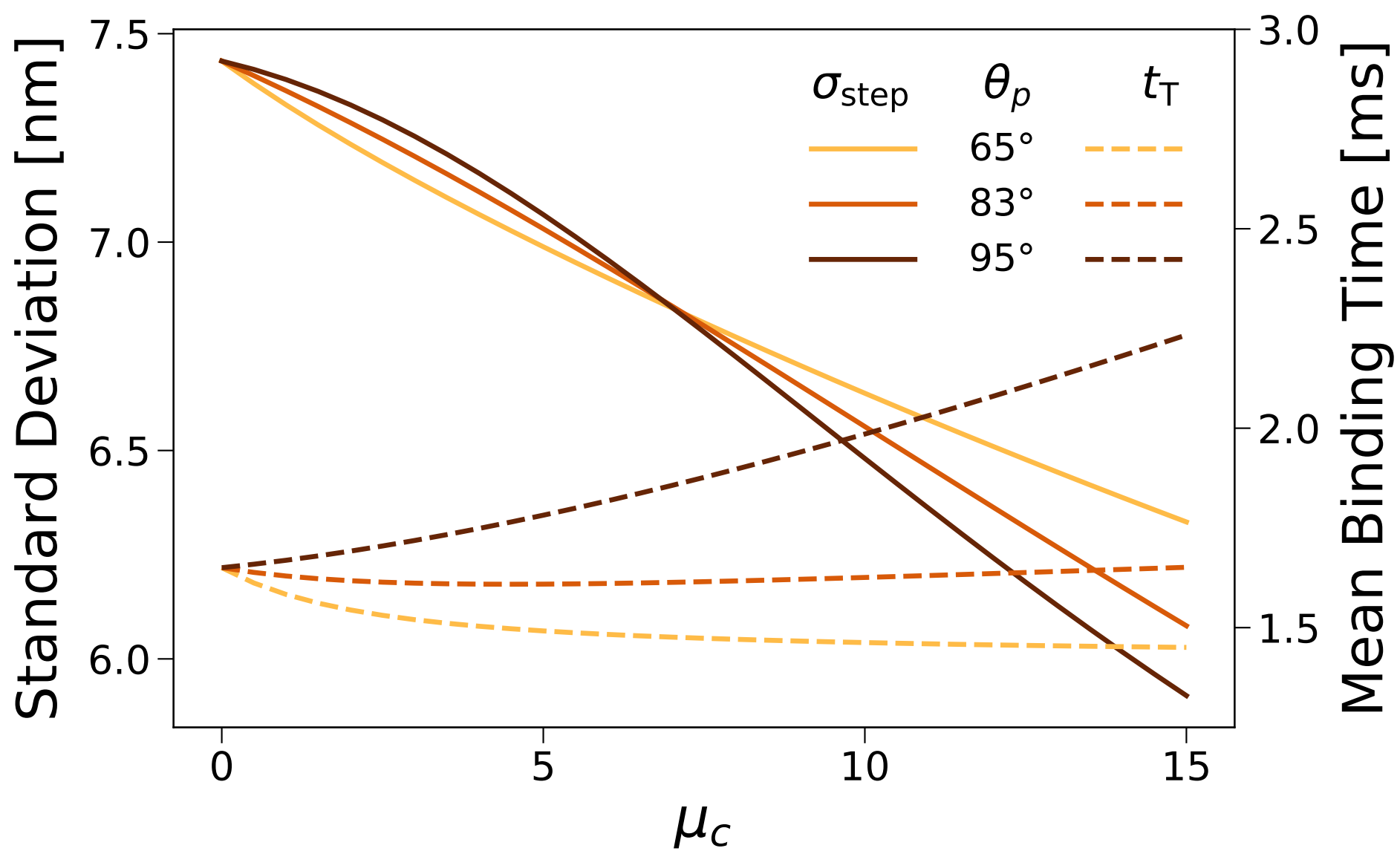

Supplement: Source data 1. [file elife-51569-data1.zip › myosin_elife_source_data/Figure 7/constraint_significance.pdf]

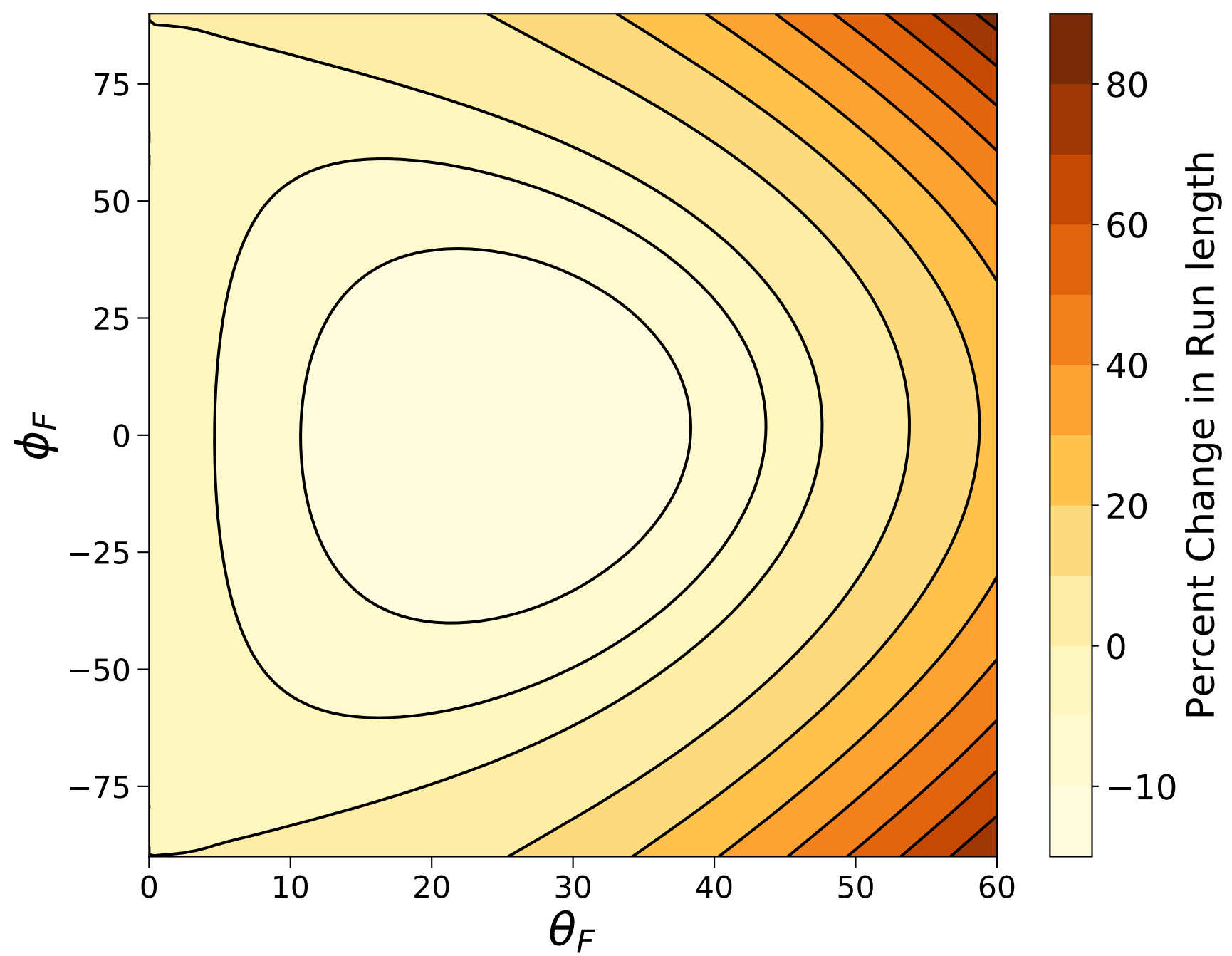

Supplement: Source data 1. [file elife-51569-data1.zip › myosin_elife_source_data/Figure 9/run_length_off_axis.pdf]

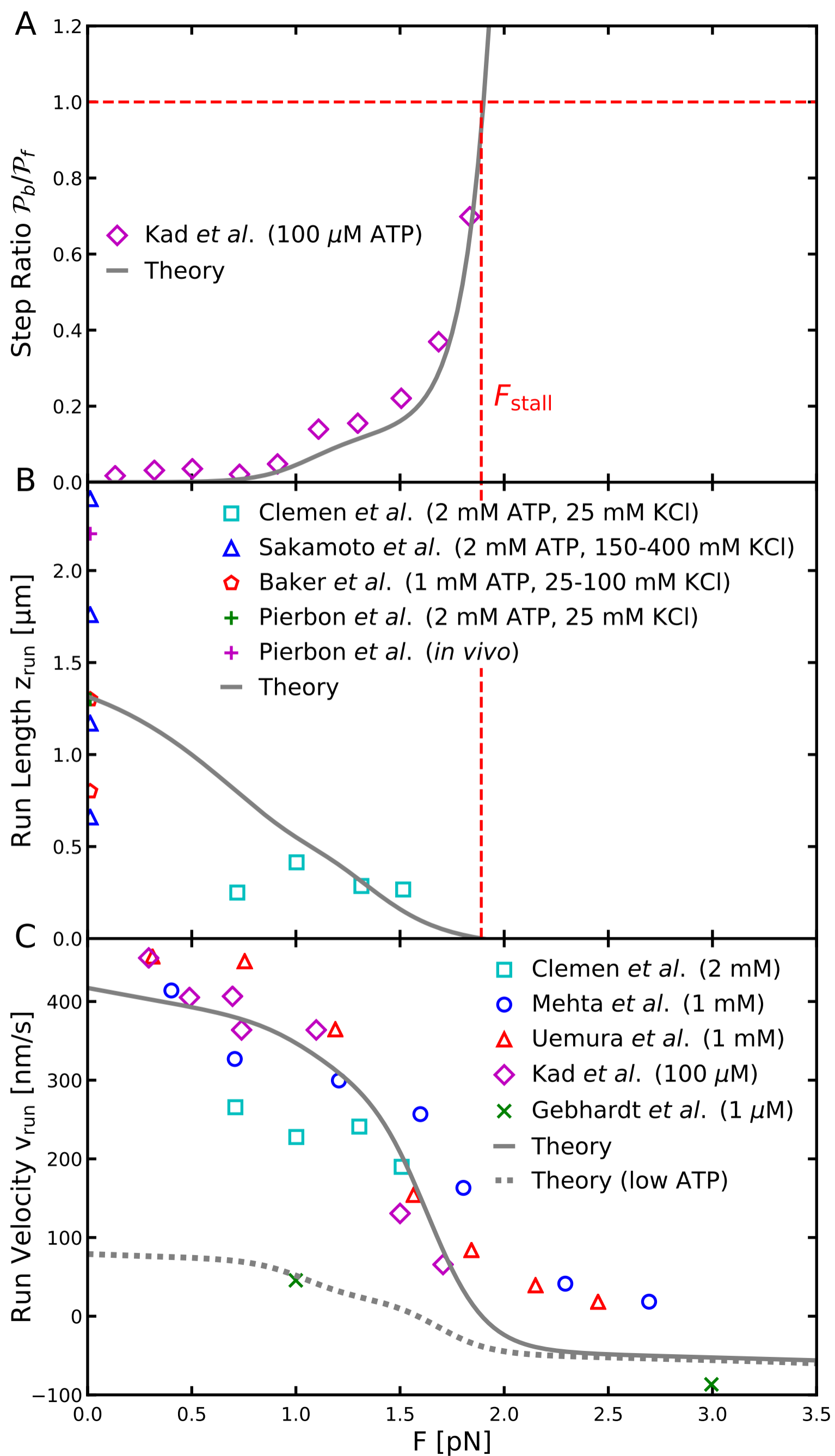

Supplement: Source data 1. [file elife-51569-data1.zip › myosin_elife_source_data/Figure 8/force_dependence_free_diffusion.pdf]

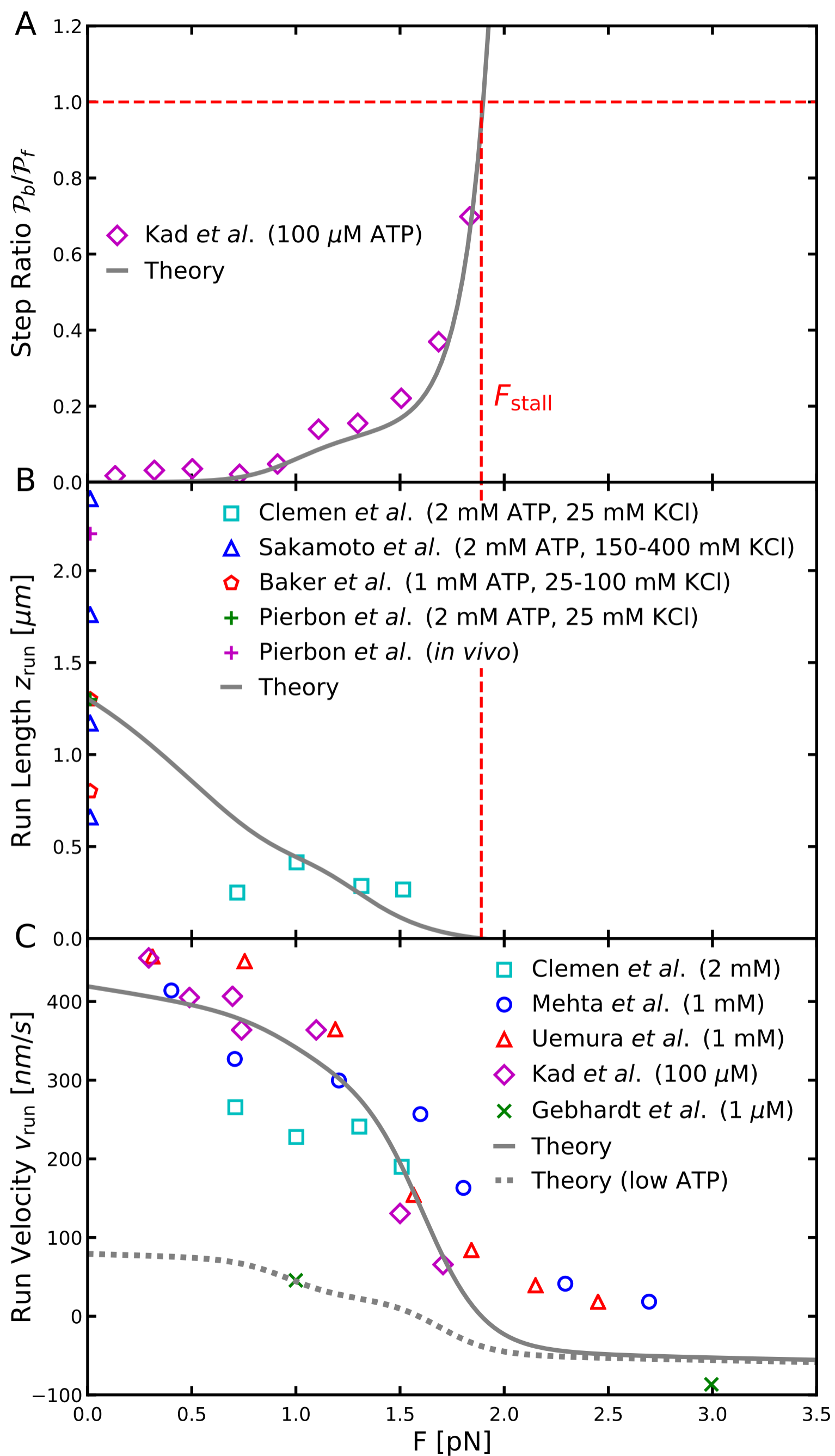

Supplement: Source data 1. [file elife-51569-data1.zip › myosin_elife_source_data/Figure 8/force_dependence.pdf]

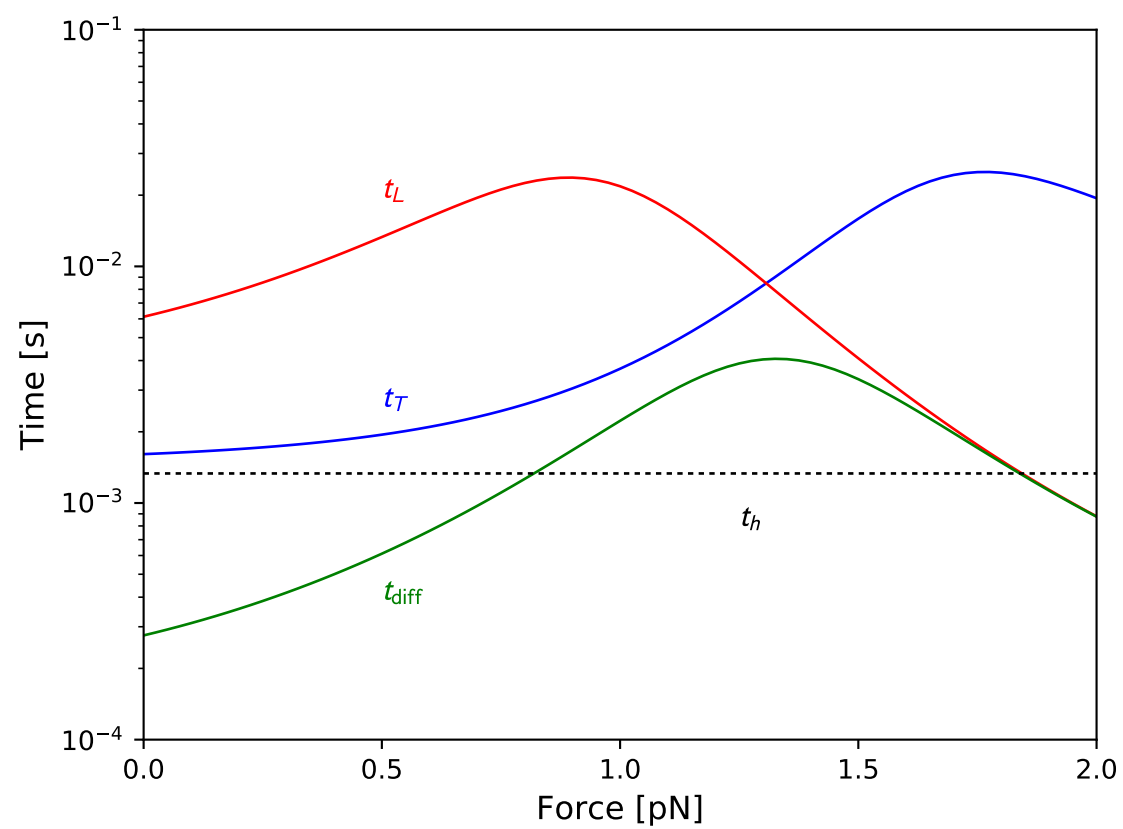

Supplement: Source data 1. [file elife-51569-data1.zip › myosin_elife_source_data/Figure 6/myosin_binding_times.pdf]

## Raw step distributions, $\mathcal{P}_{\text{dist}}^n$ :

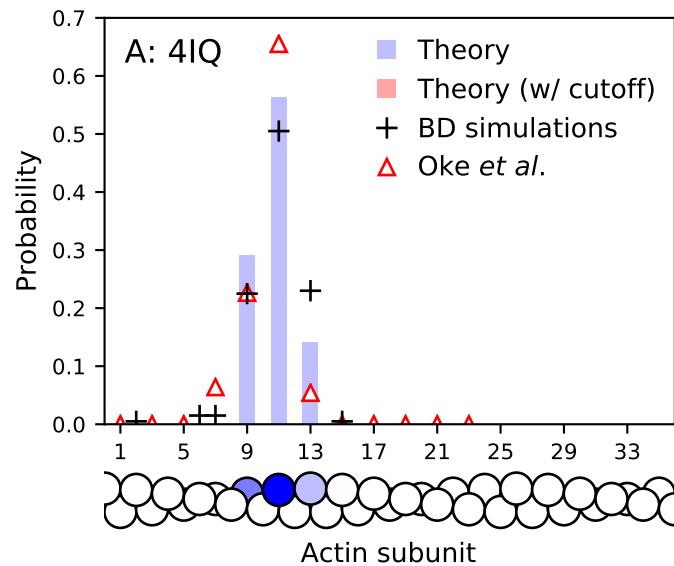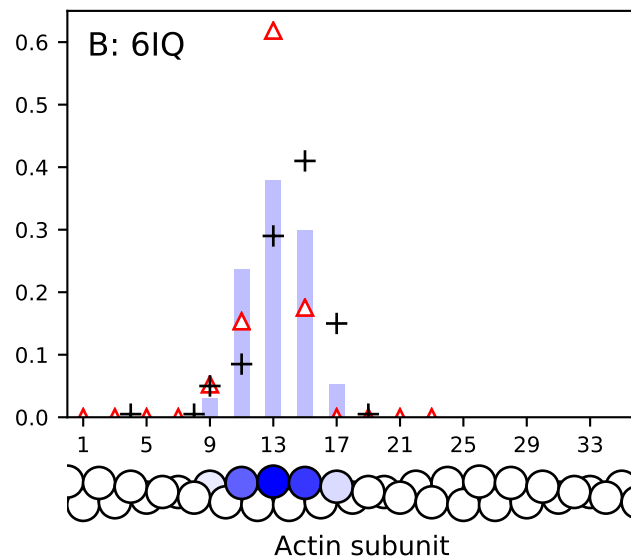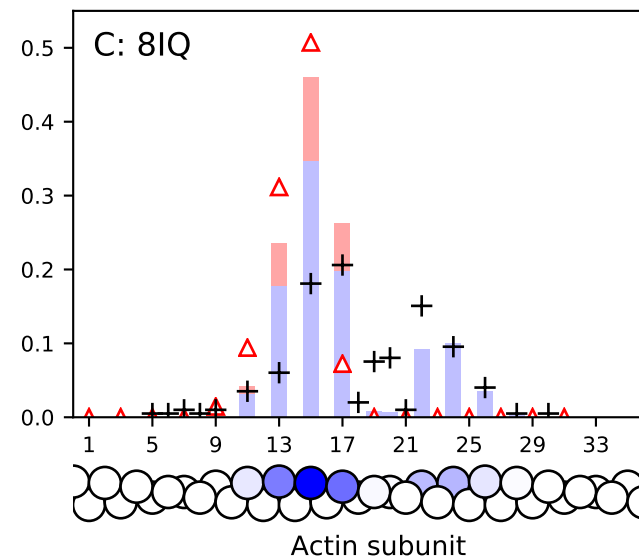

## Full step distributions, $\mathcal{P}_{\text{T}}(z_n)$ :

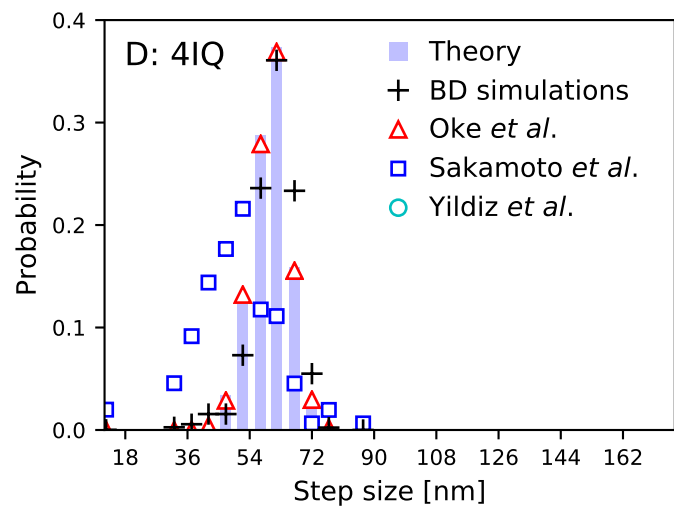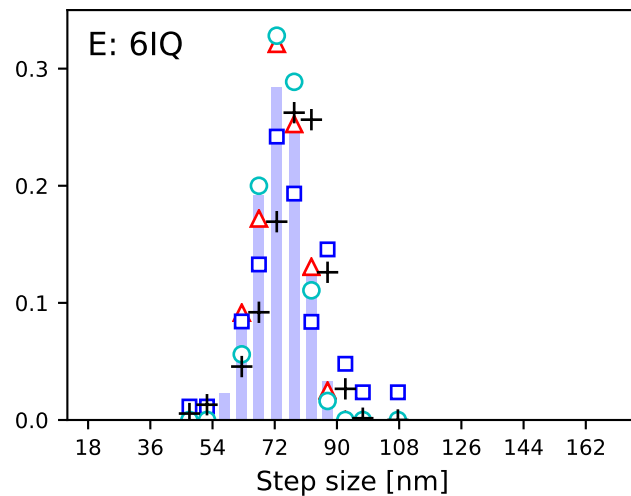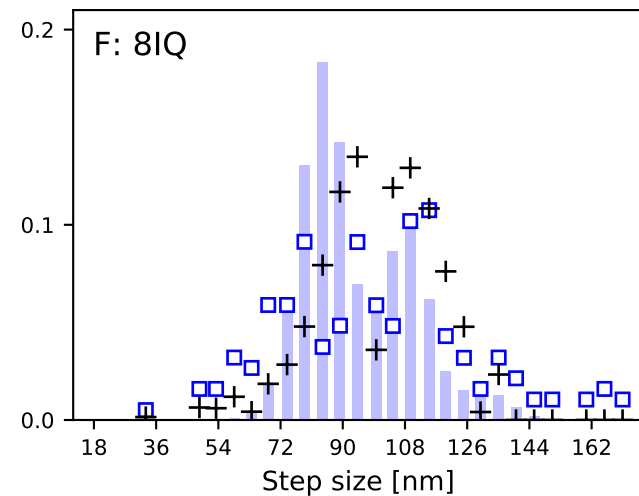

Supplement: Source data 1. [file elife-51569-data1.zip › myosin_elife_source_data/Figure 4/step_distributions.pdf]

## Raw step distributions, $\mathcal{P}_{\text{dist}}^n$ :

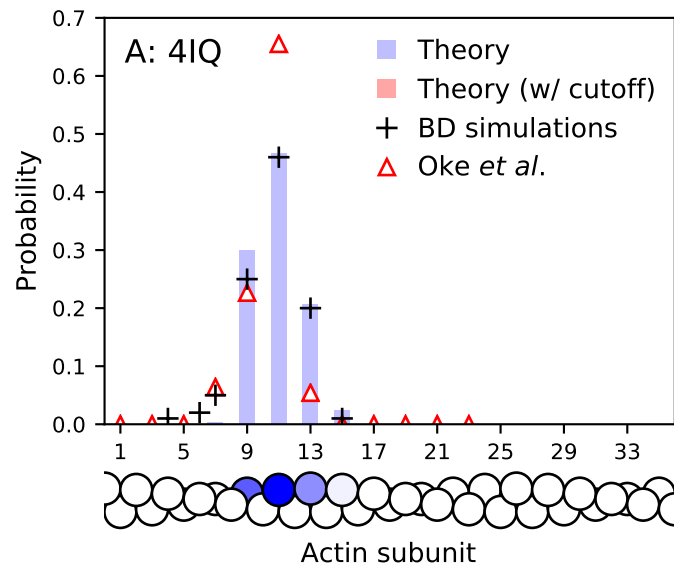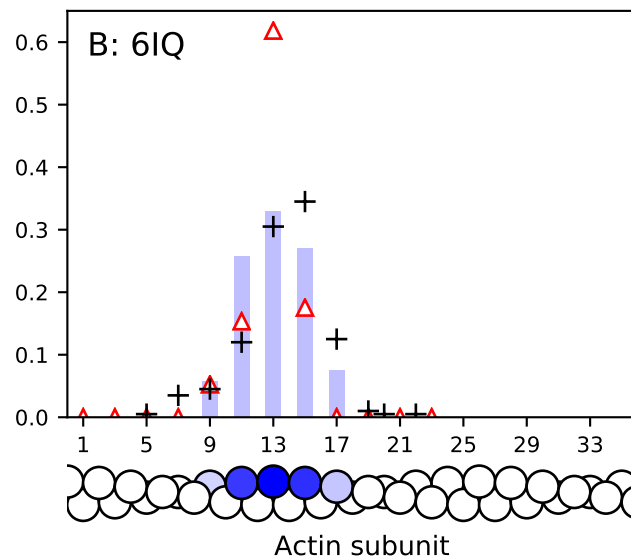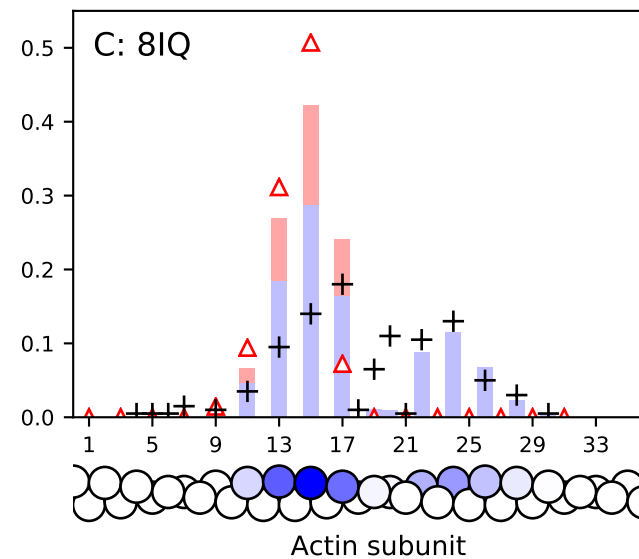

## Full step distributions, $\mathcal{P}_{\text{T}}(z_n)$ :

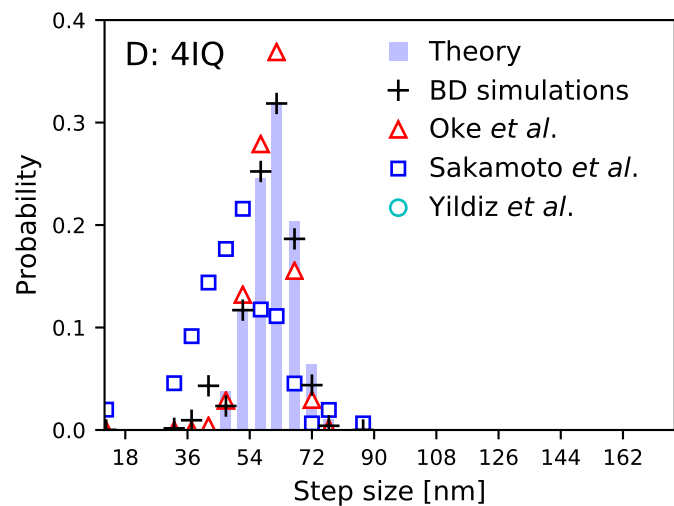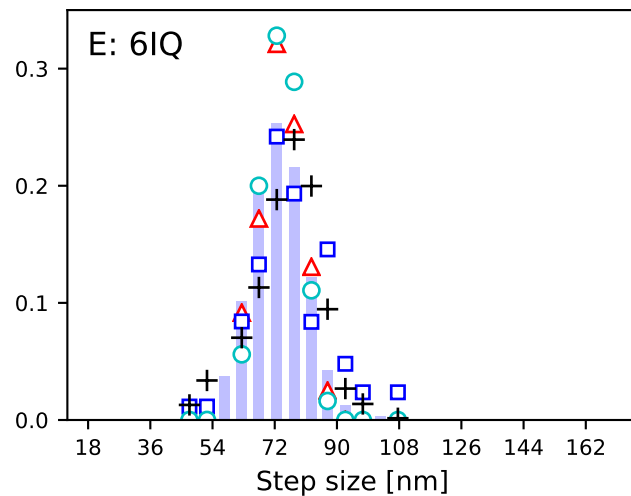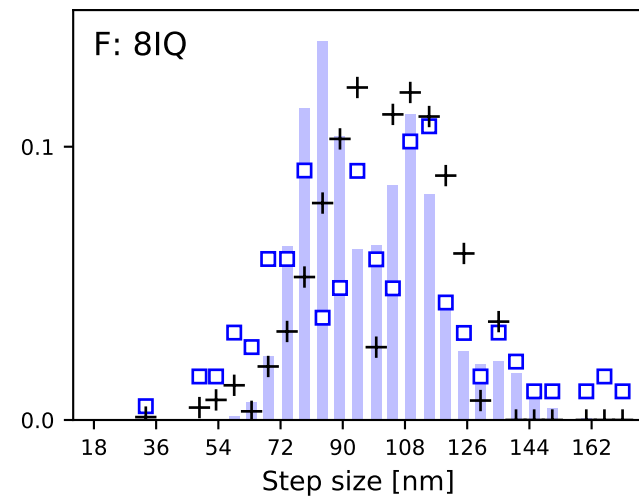

Supplement: Source data 1. [file elife-51569-data1.zip › myosin_elife_source_data/Figure 4/step_distributions_free_diffusion.pdf]

# Analytical Theory

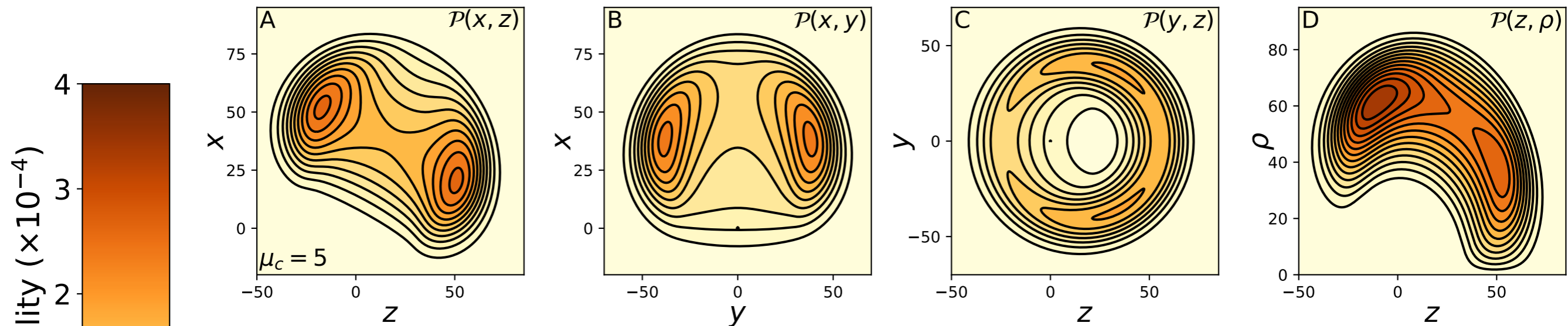

# Brownian Dynamics Simulations

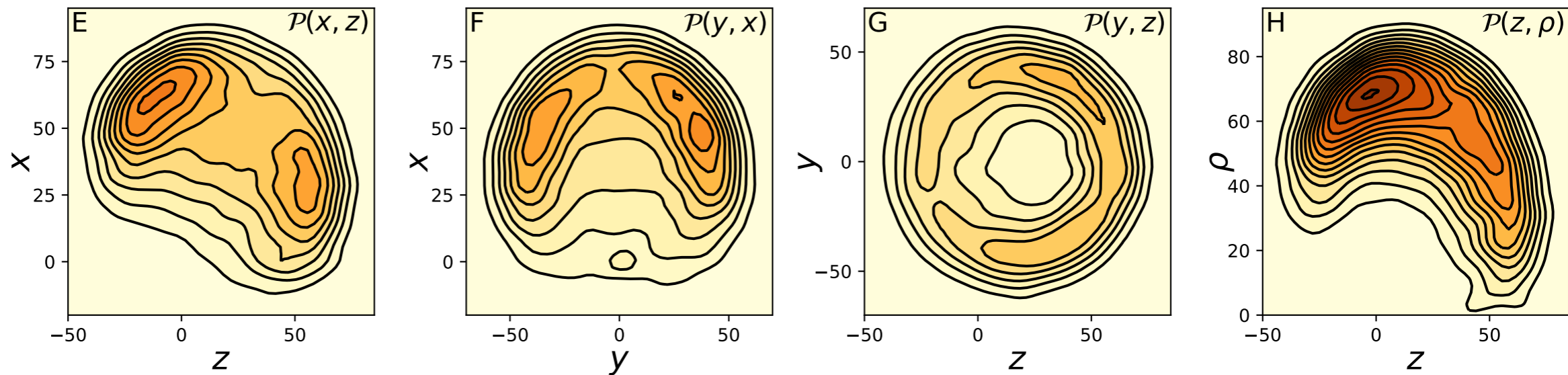

Supplement: Source data 1. [file elife-51569-data1.zip › myosin_elife_source_data/Figure 3/diffusion_figure_8IQ.pdf]

# Analytical Theory

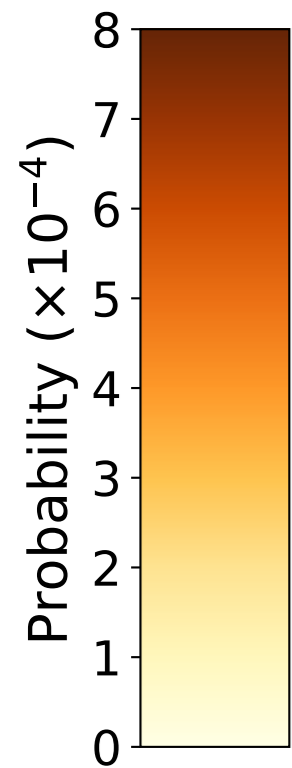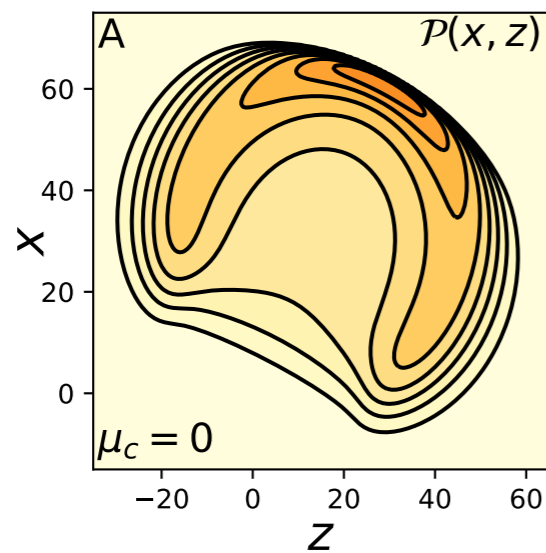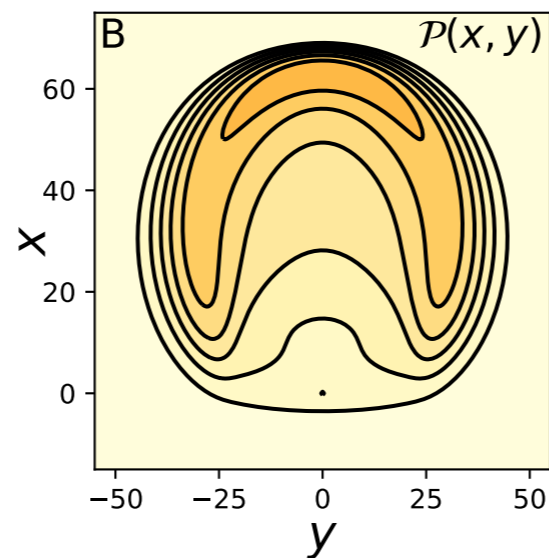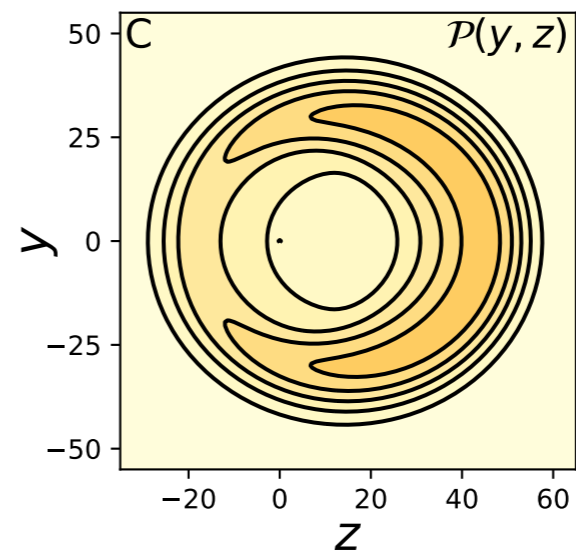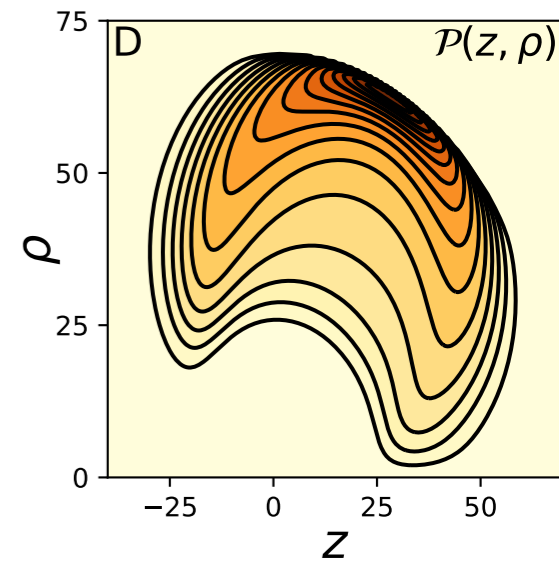

# Brownian Dynamics Simulations

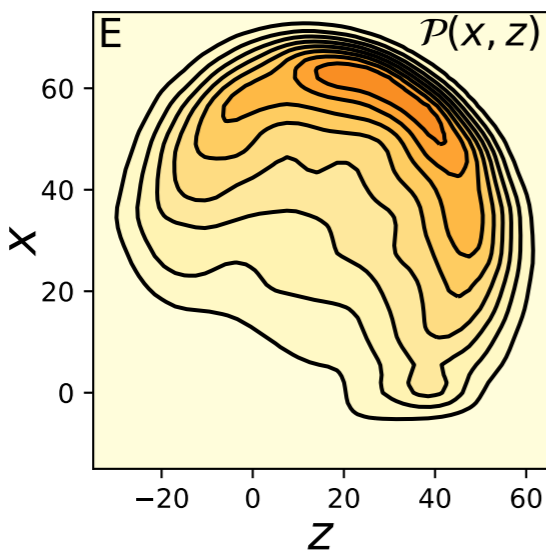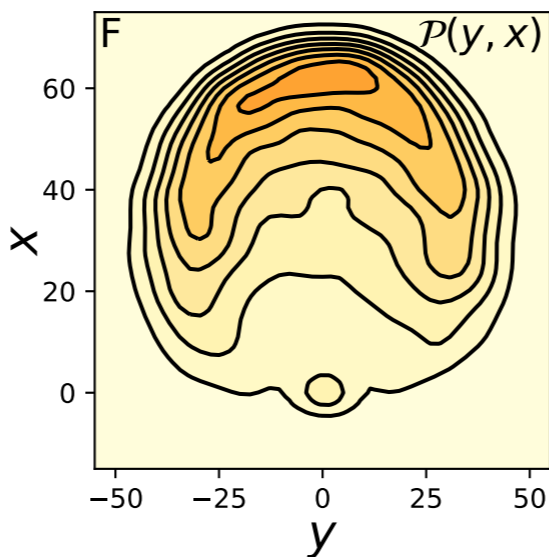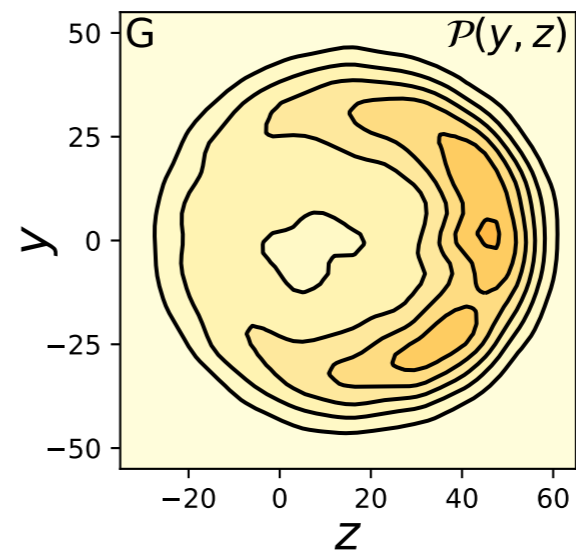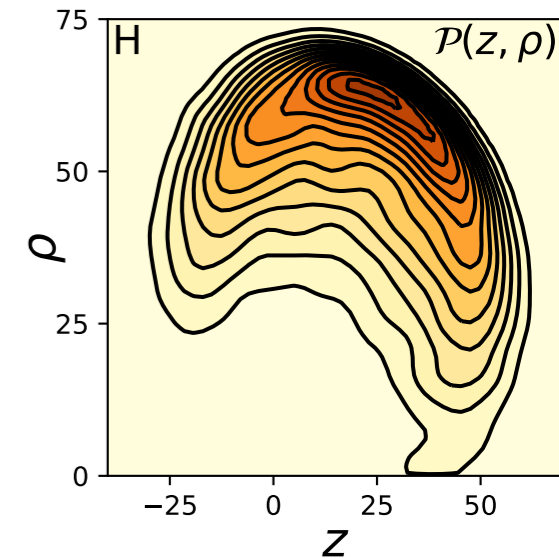

Supplement: Source data 1. [file elife-51569-data1.zip › myosin_elife_source_data/Figure 3/diffusion_figure_free_alt_projections.pdf]

# Analytical Theory

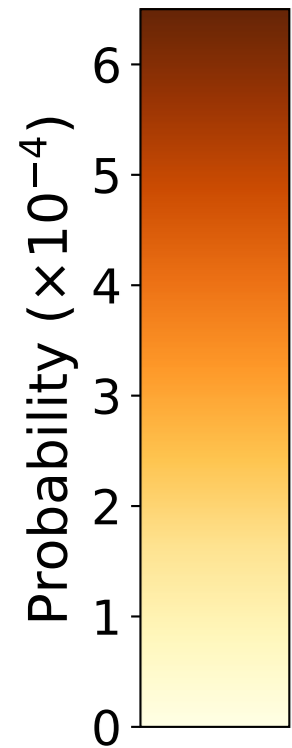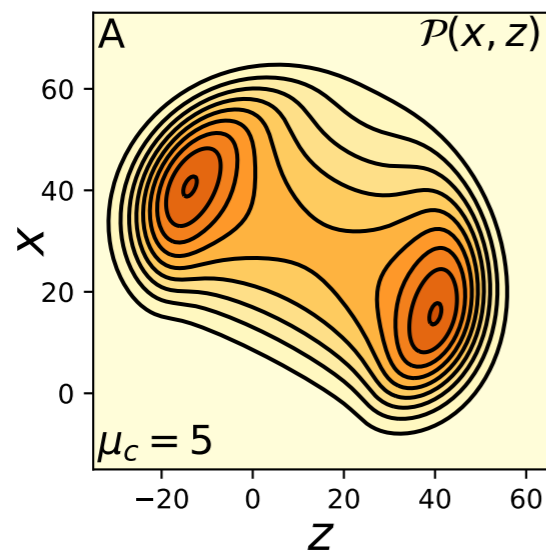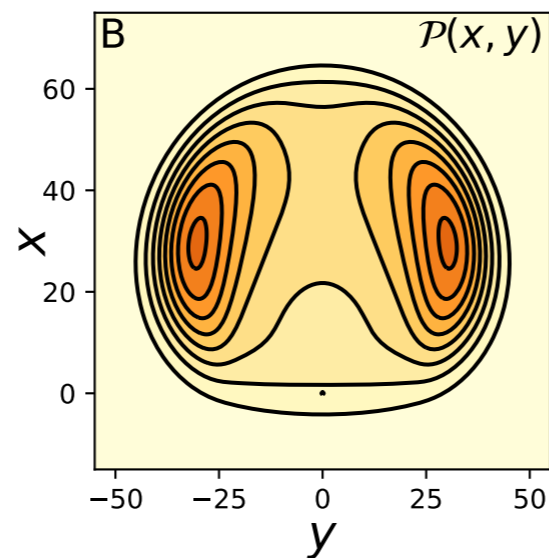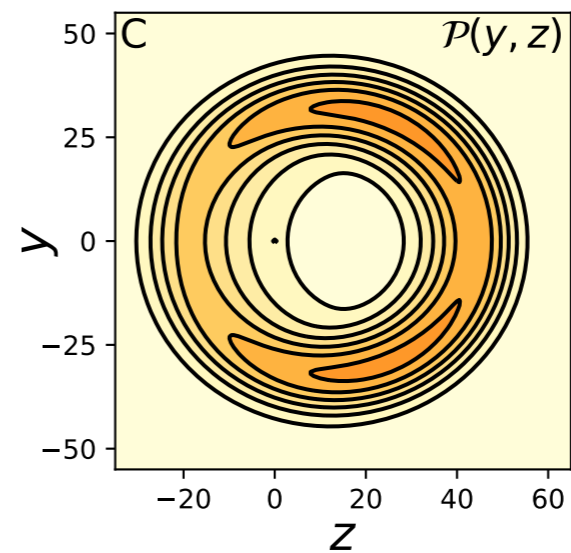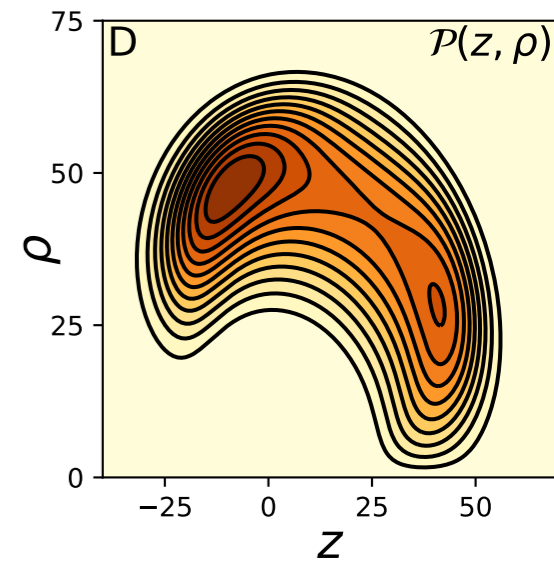

# Brownian Dynamics Simulations

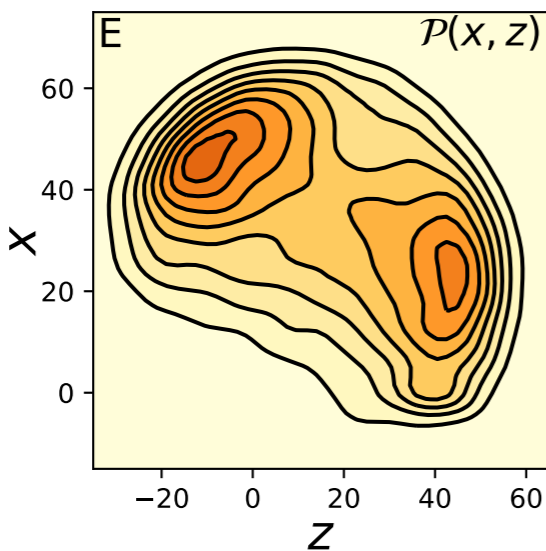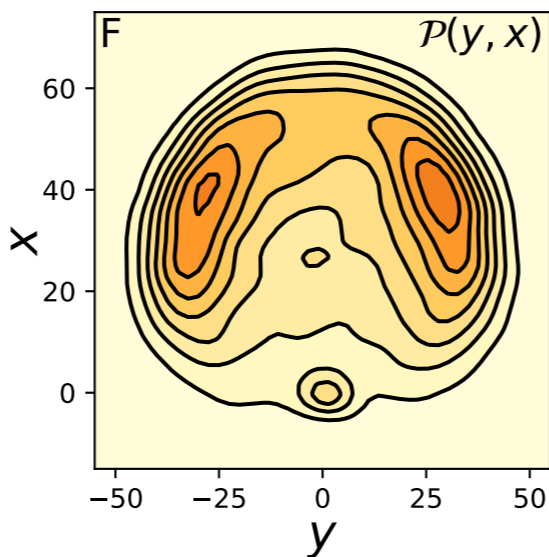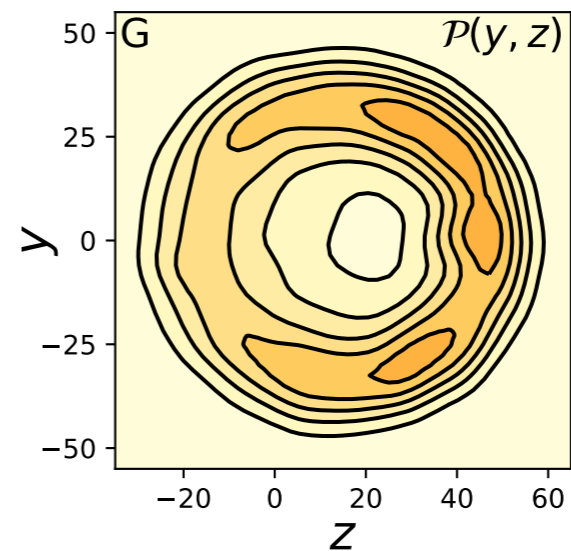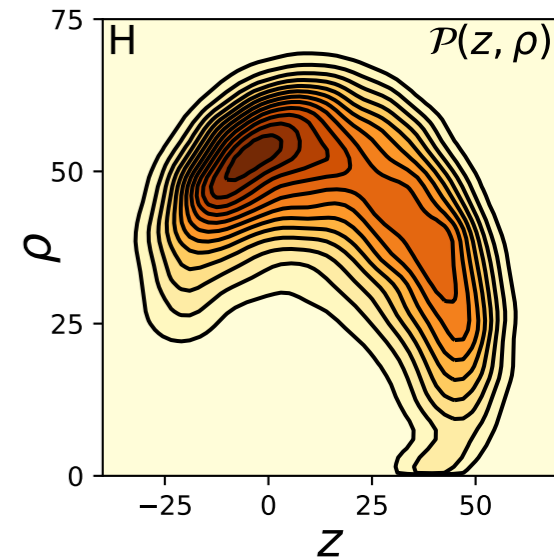

Supplement: Source data 1. [file elife-51569-data1.zip › myosin_elife_source_data/Figure 3/diffusion_figure_constrained_alt_projections.pdf]

## Free Diffusion ( $\mu_c = 0$ )

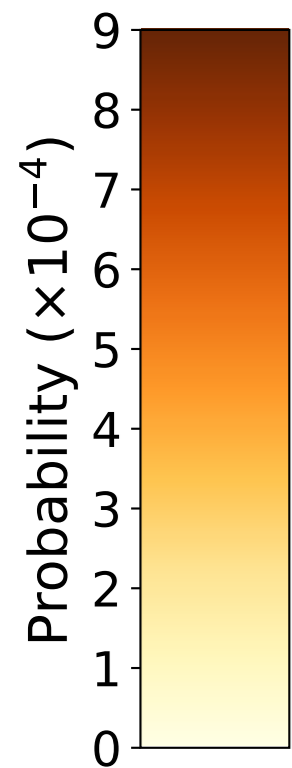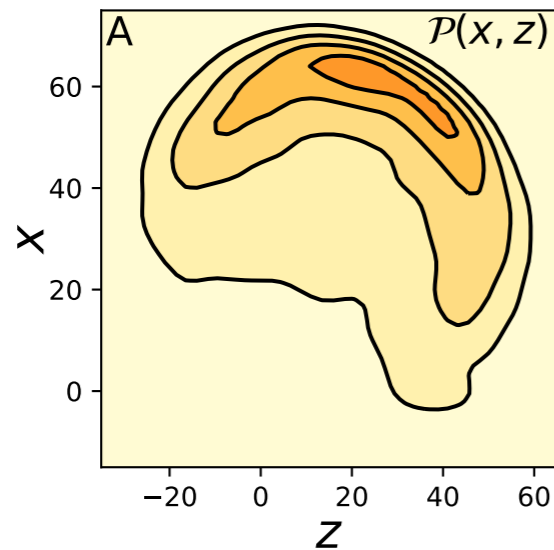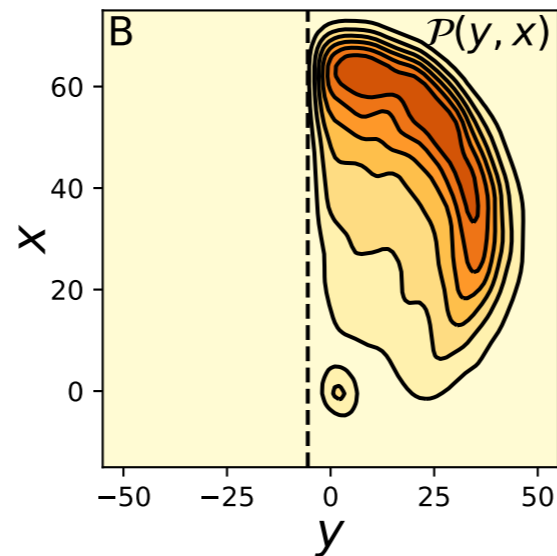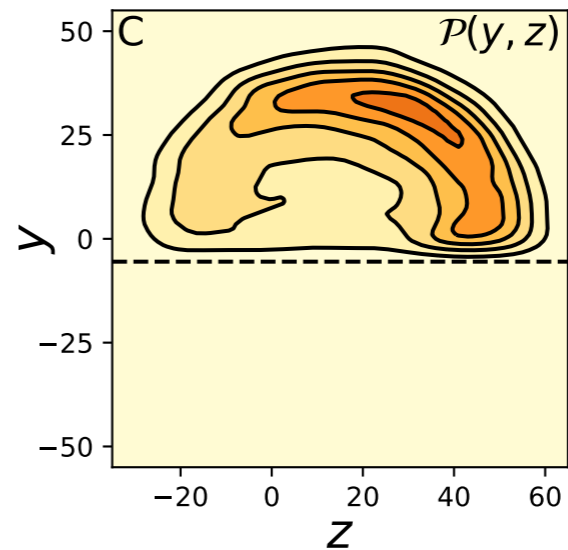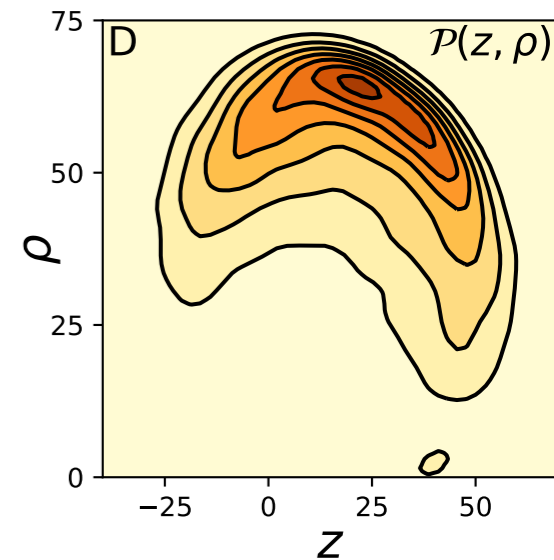

## Constrained Diffusion ( $\mu_c = 5$ )

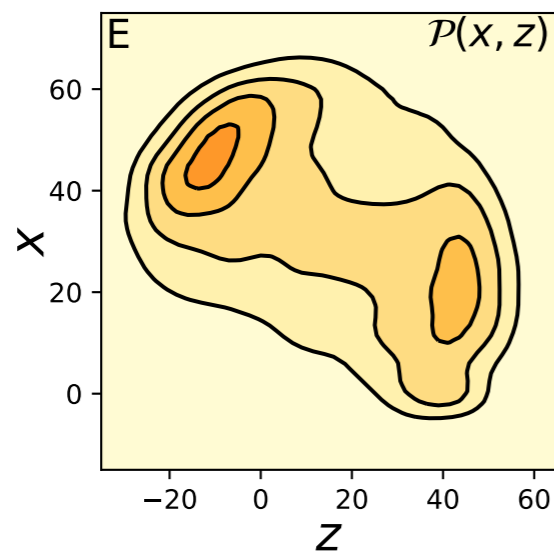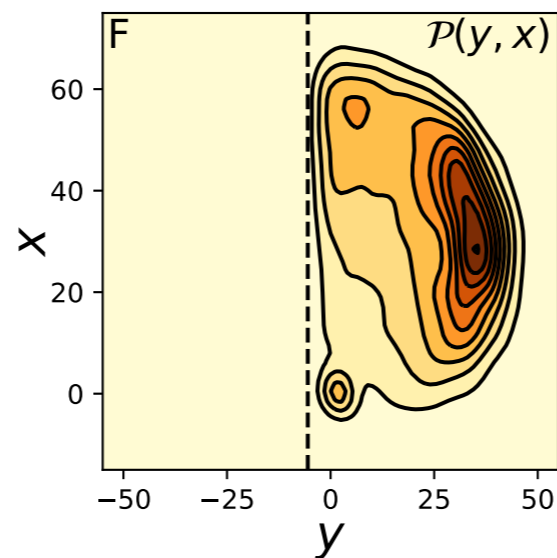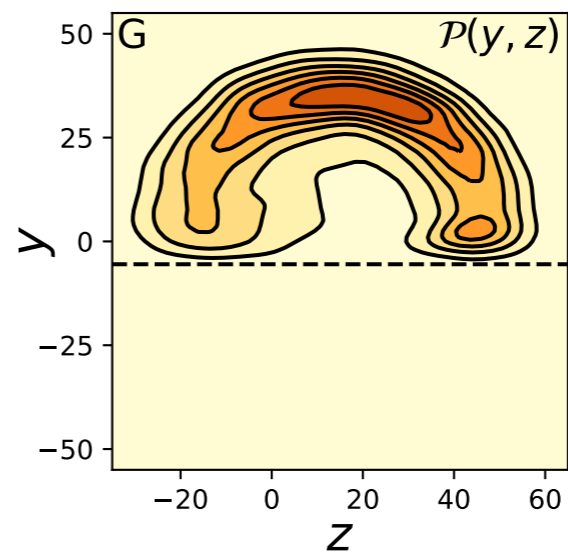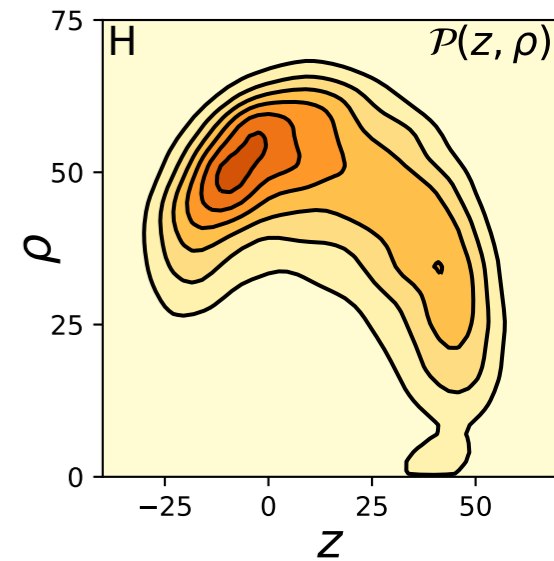

Supplement: Source data 1. [file elife-51569-data1.zip › myosin_elife_source_data/Figure 3/diffusion_figure_volume_exclusion.pdf]

# Analytical Theory

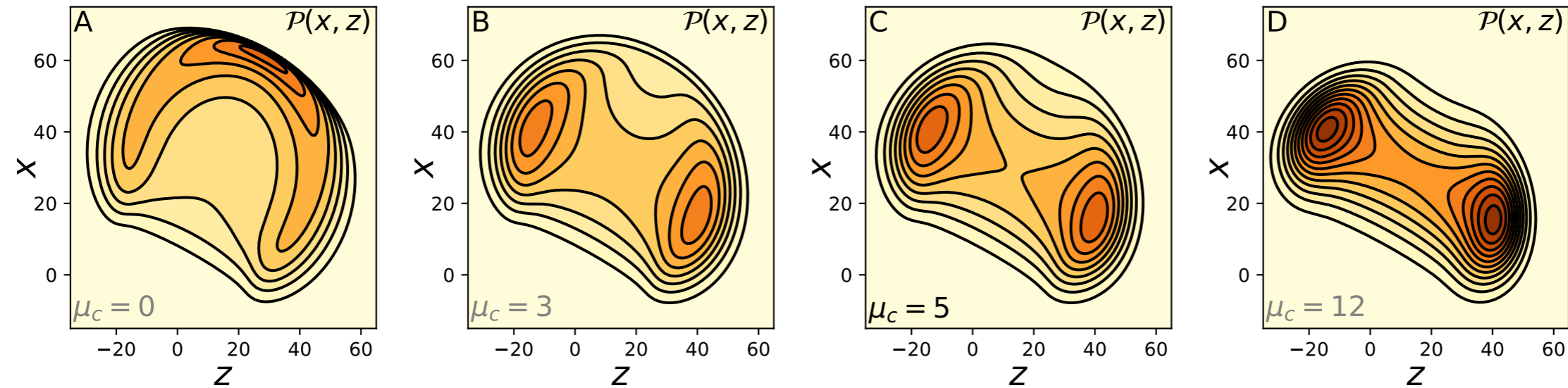

# Brownian Dynamics Simulations

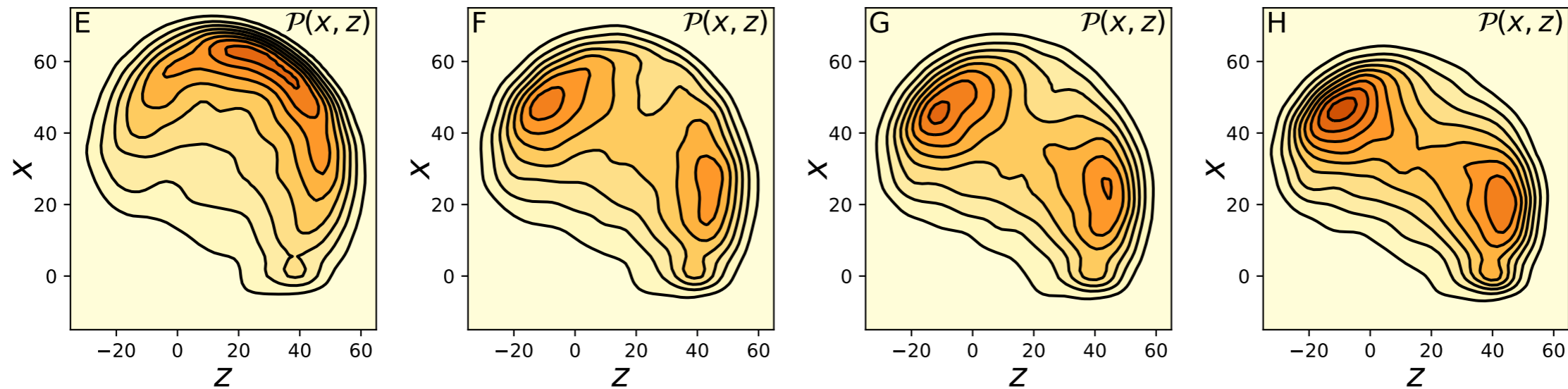

# Experiment

(Andrecka et al., 2015)

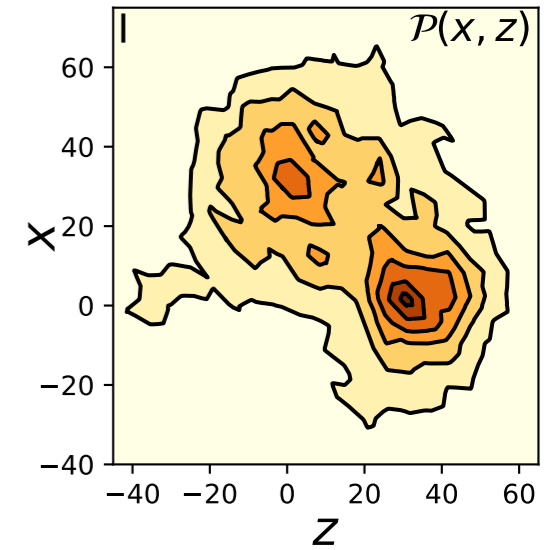

Supplement: Source data 1. [file elife-51569-data1.zip › myosin_elife_source_data/Figure 3/diffusion_figure.pdf]

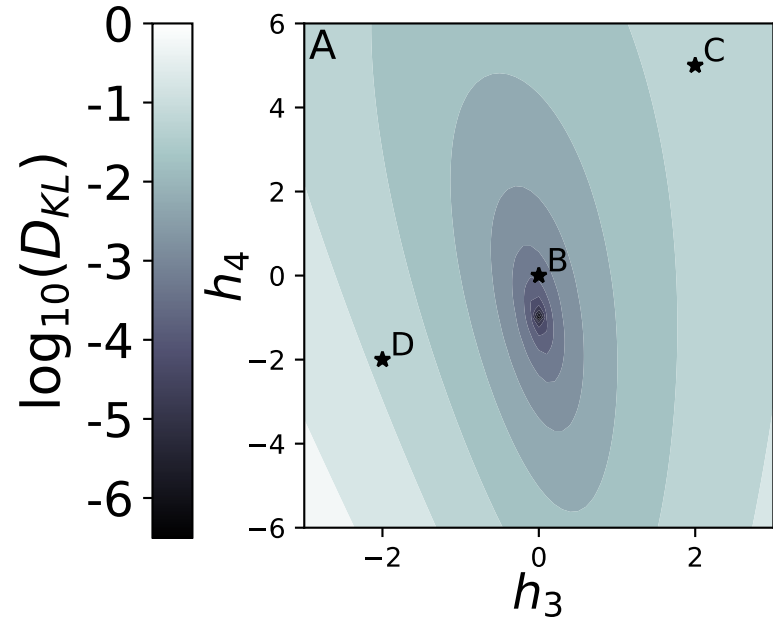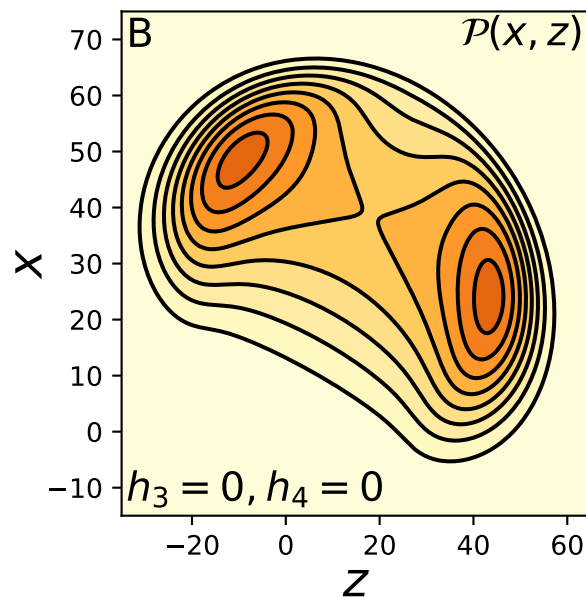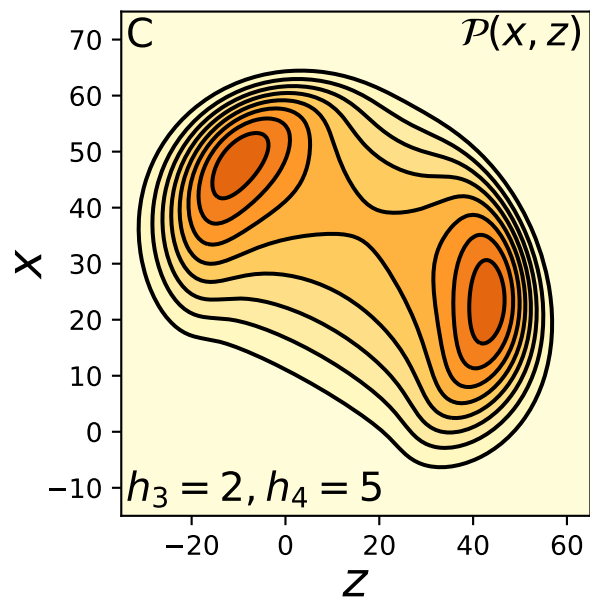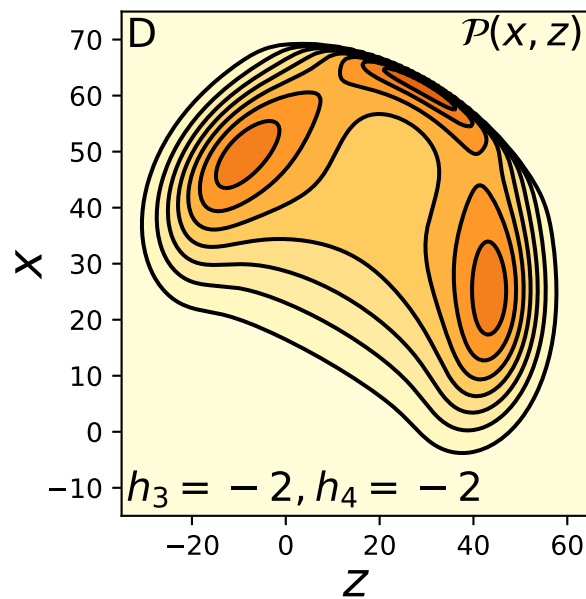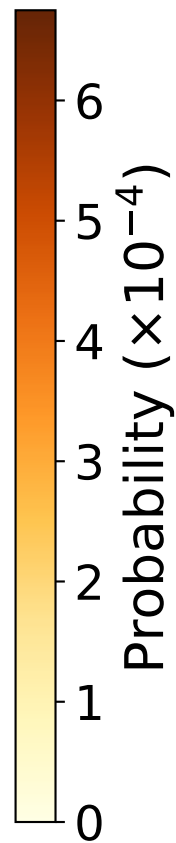

Supplement: Source data 1. [file elife-51569-data1.zip › myosin_elife_source_data/Figure 3/diffusion_figure_alt_potentials.pdf]

# Analytical Theory

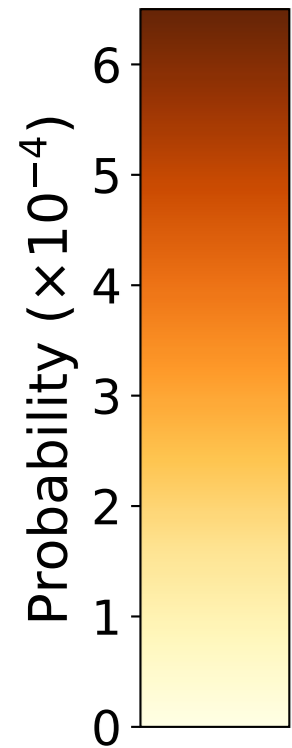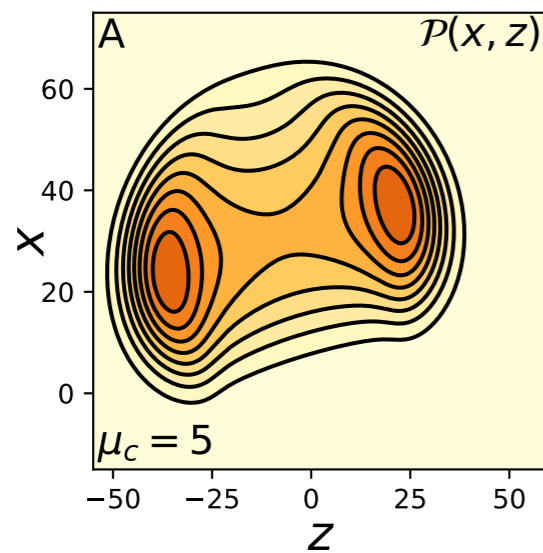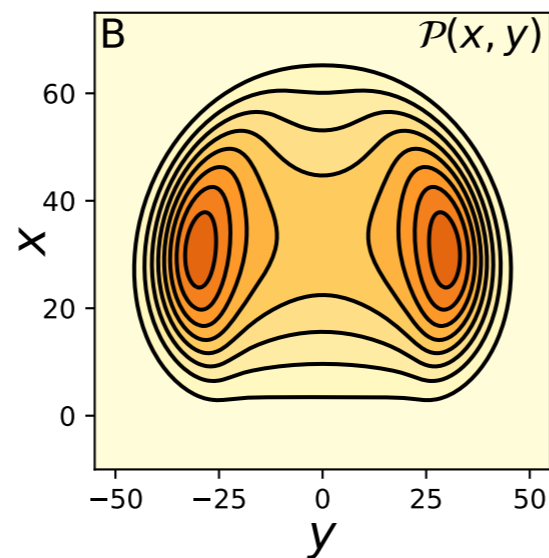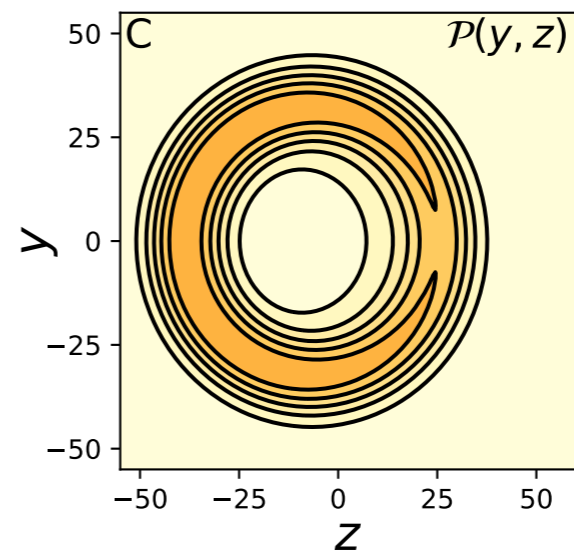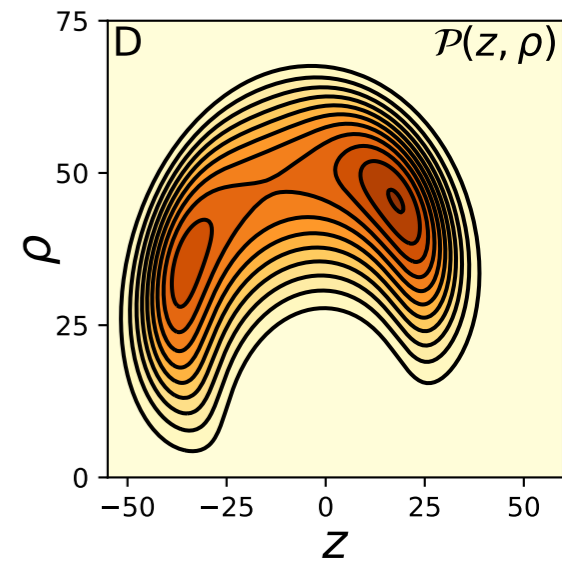

# Brownian Dynamics Simulations

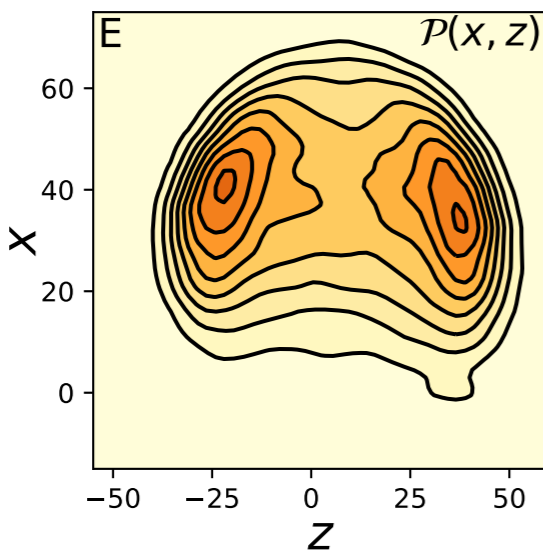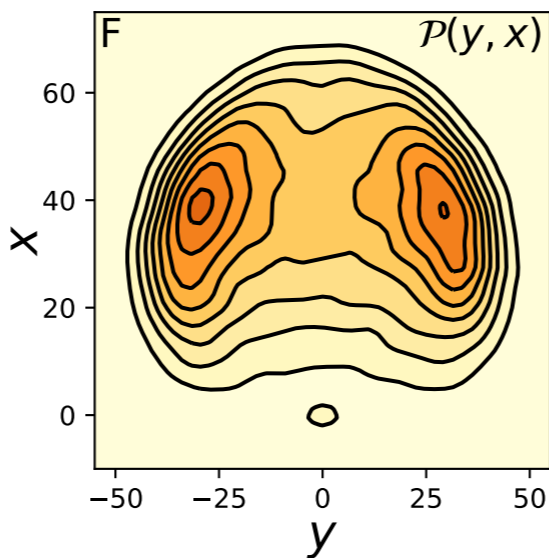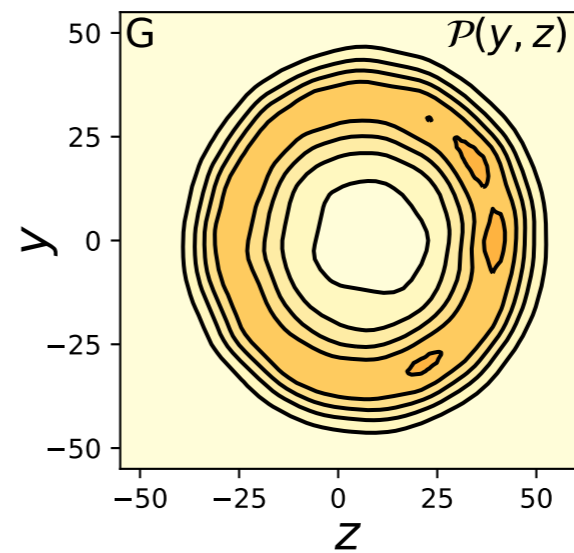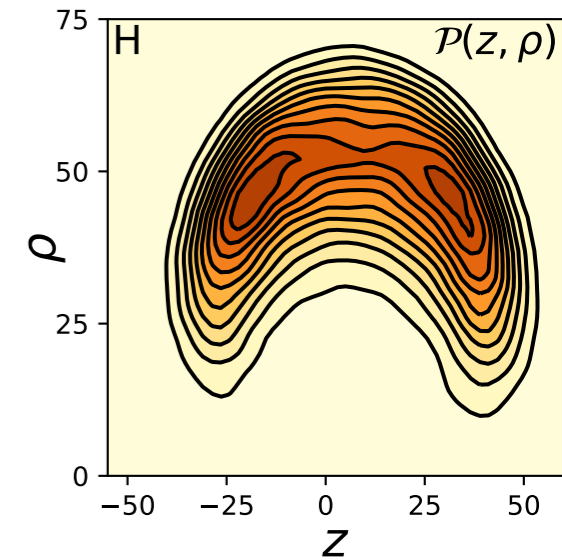

Supplement: Source data 1. [file elife-51569-data1.zip › myosin_elife_source_data/Figure 3/diffusion_figure_force_dependence.pdf]

# Analytical Theory

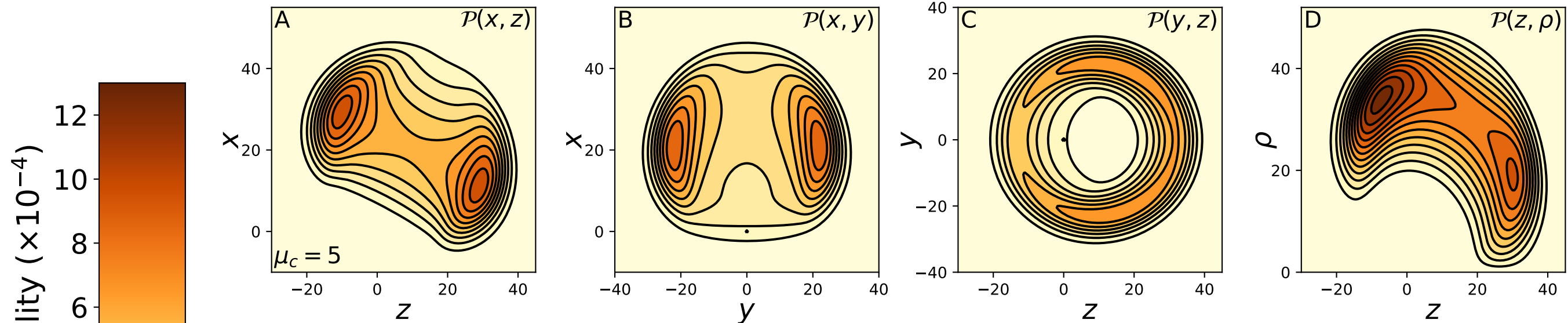

# Brownian Dynamics Simulations

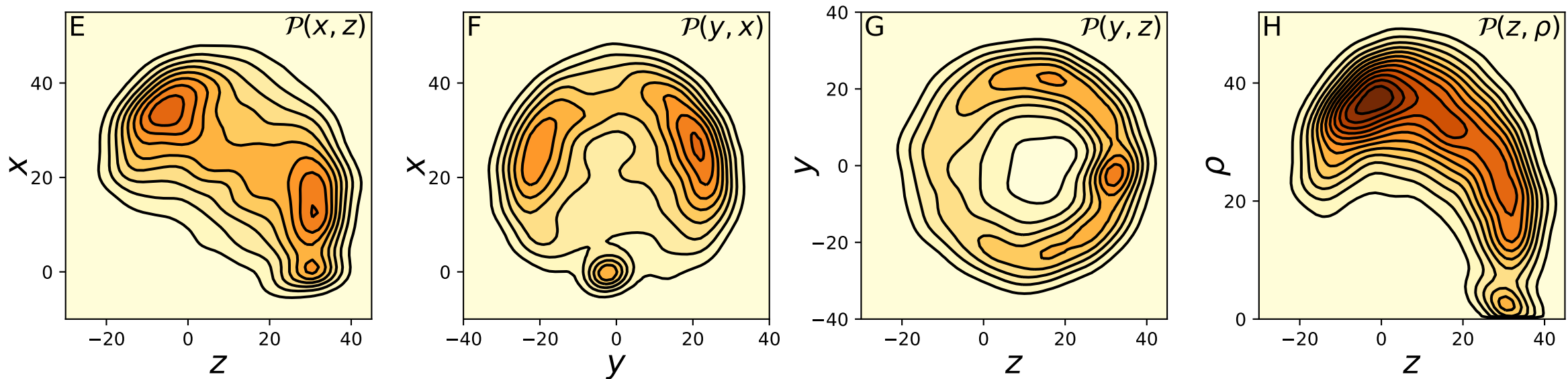

Supplement: Source data 1. [file elife-51569-data1.zip › myosin_elife_source_data/Figure 3/diffusion_figure_4IQ.pdf]

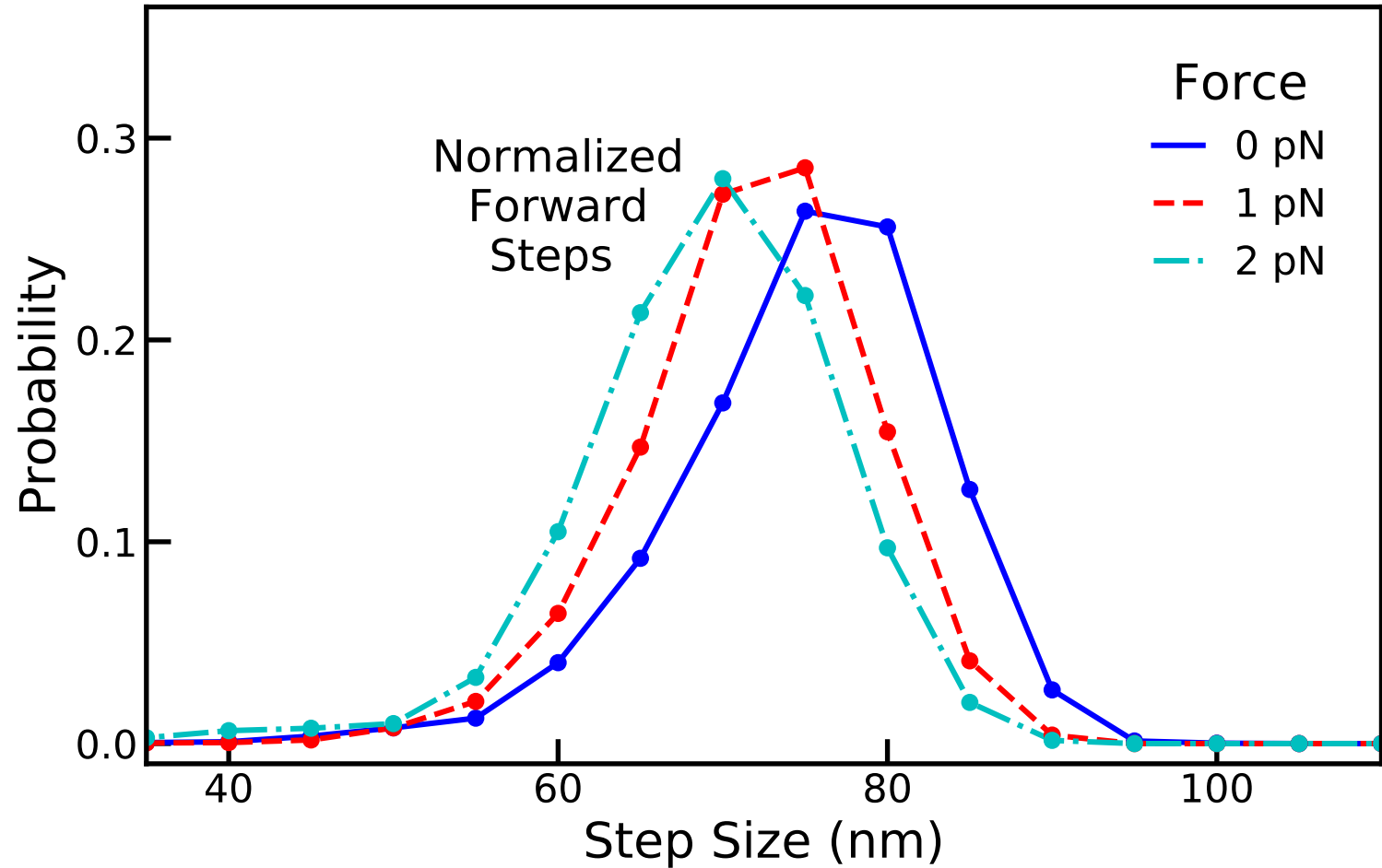

Supplement: Source data 1. [file elife-51569-data1.zip › myosin_elife_source_data/Figure 5/force_step_distributions_BD.pdf]

A

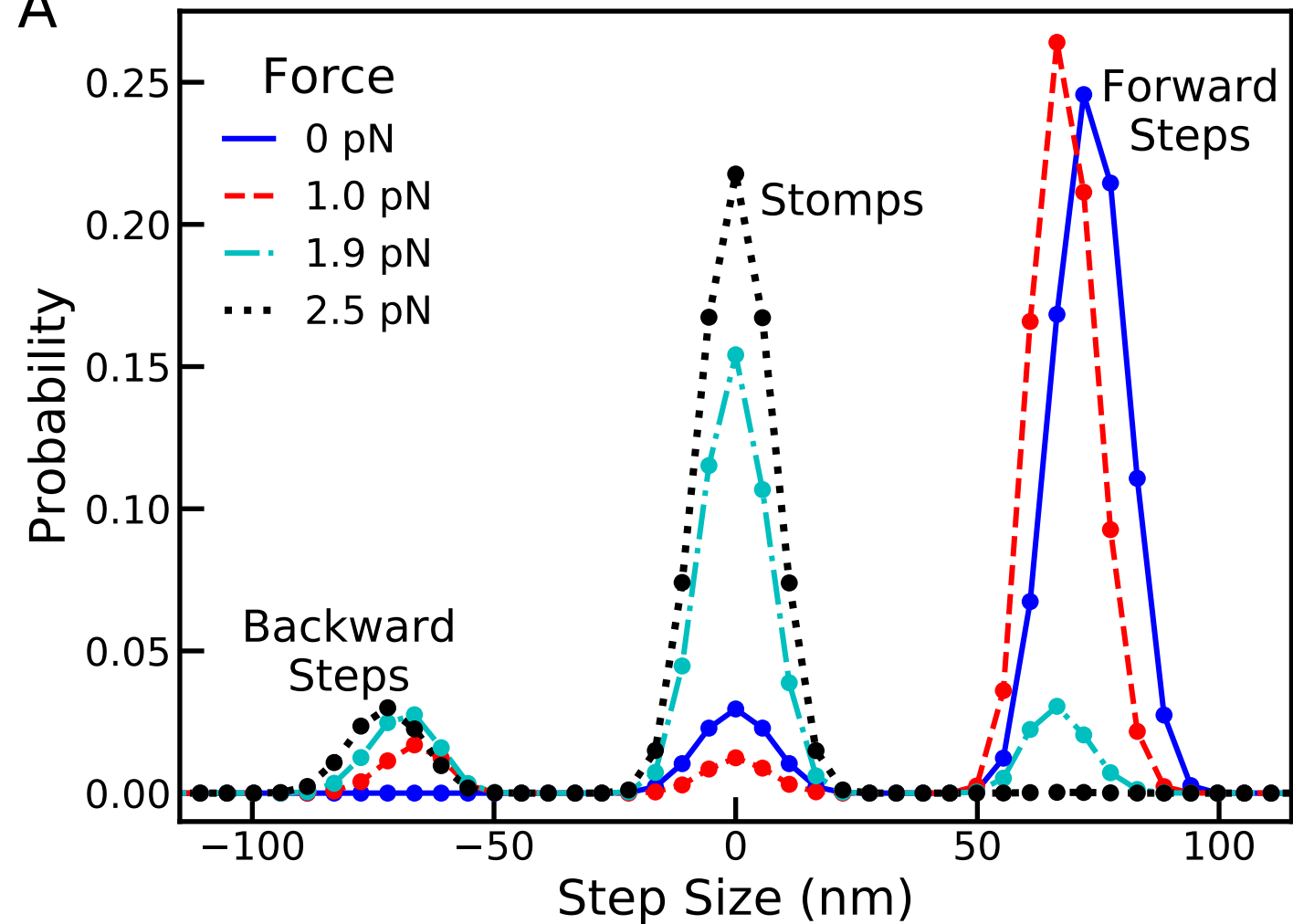

B

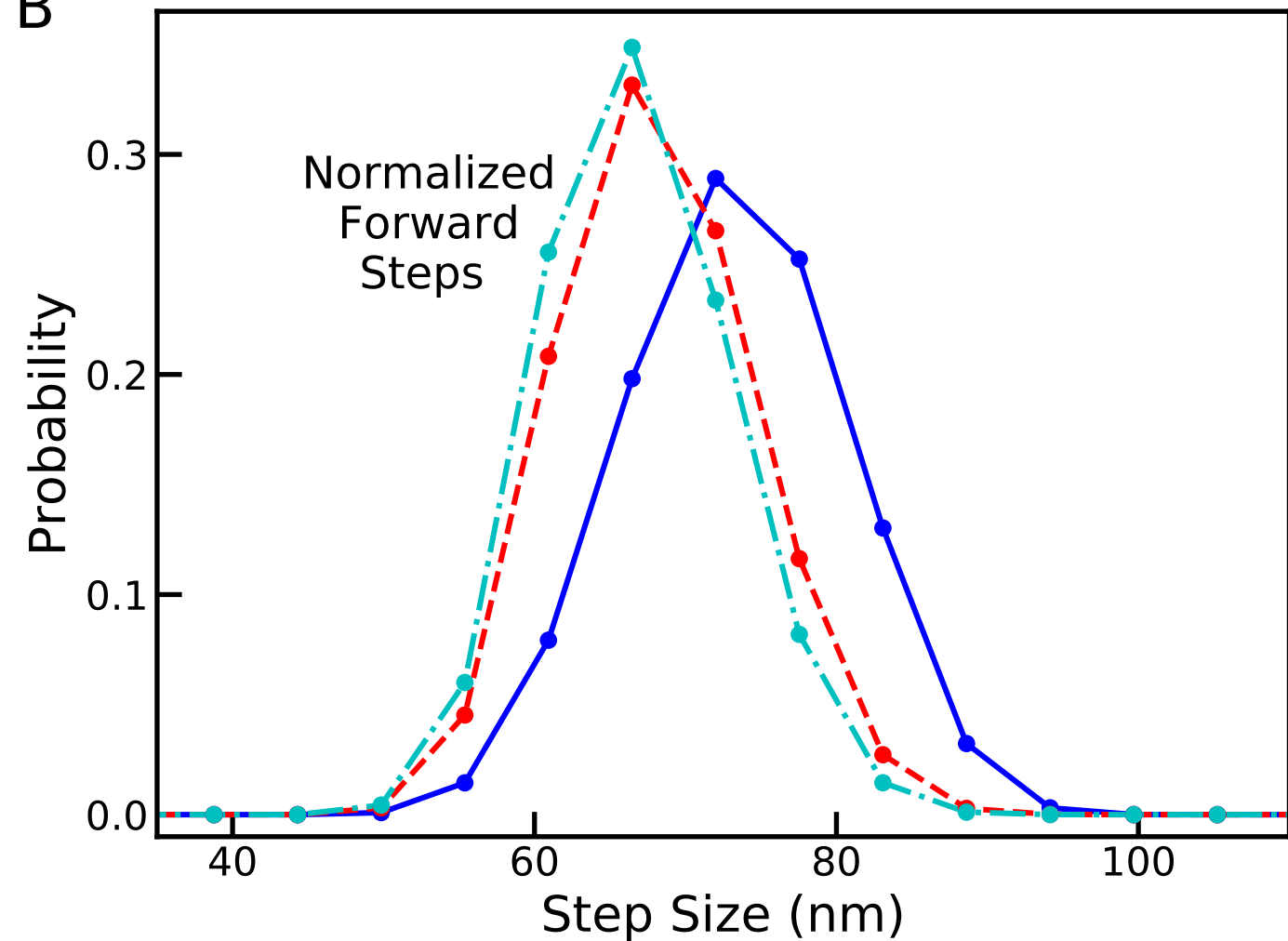

Supplement: Source data 1. [file elife-51569-data1.zip › myosin_elife_source_data/Figure 5/force_step_distributions.pdf]
